# PROSSTT: probabilistic simulation of single-cell RNA-seq data for complex differentiation processes

**DOI:** 10.1093/bioinformatics/btz078

**Published:** 2019-02-01

**Authors:** Nikolaos Papadopoulos, Parra R Gonzalo, Johannes Söding

**Affiliations:** Quantitative and Computational Biology, Max Planck Institute for Biophysical Chemistry, Göttingen, Germany

## Abstract

**Summary:**

Cellular lineage trees can be derived from single-cell RNA sequencing snapshots of differentiating cells. Currently, only datasets with simple topologies are available. To test and further develop tools for lineage tree reconstruction, we need test datasets with known complex topologies. PROSSTT can simulate scRNA-seq datasets for differentiation processes with lineage trees of any desired complexity, noise level, noise model and size. PROSSTT also provides scripts to quantify the quality of predicted lineage trees.

**Availability and implementation:**

https://github.com/soedinglab/prosstt.

**Supplementary information:**

Supplementary data are available at *Bioinformatics* online.

## 1 Introduction

Recent advances in single-cell RNA sequencing (scRNA-seq) (Klein *et al.*, 2015; Macosko, 2015) make it possible to generate expression profiles for thousands of cells. Clustering the transcriptomic snapshot of a cell population reveals cell types (Trapnell, 2015), and ordering the cells according to their progress through differentiation reconstructs cellular lineage trees, offering insights into czomplex processes such as organogenesis (Camp *et al.*, 2017). The change in gene expression along the reconstructed trees gives us unprecedented, time-resolved data to quantitatively investigate the gene regulatory processes underlying cellular development.

As more and more complex processes are investigated, there will be a need to derive lineage trees of topologies more complex than linear or singly-branched ones. Also, with various methods already published (Rostom *et al.*, 2017) and more being developed, the need to quantify method performance is becoming more pressing. With the available data, assessing method performance is challenging as there are no datasets with known ground truth, i.e. data with known intrinsic developmental time and cell identity. These needs can be addressed by simulating realistic scRNA-seq datasets of complex dynamic processes.

Tools like Splatter (Zappia *et al.*, 2017) and dyngen (Saelens *et al.*, 2018) can simulate scRNA-seq data from lineage trees, however both have limitations. In particular, Splatter does not explicitly model coordinated change in gene expression, which results in tree segments that are in truth non-adjacent being placed close to each other. This happens in gene expression space as well as after dimensionality reduction (Supplementary Section S5). Additionally, Splatter doesn’t provide a global pseudotime for the simulated cells, reducing its usefulness in the context of the evaluation of tree inference methods. Dyngen is built around a gene regulatory network that gives rise to a certain network topology. This requires users to design the regulatory network or use one of the pre-generated modules, which limits the complexity of the topologies that can be simulated.

Here we present PROSSTT (PRObabilistic Simulation of Single-cell RNA-seq Tree-like Topologies), a python package for simulating UMI counts from scRNA-seq experiements of complex differentiation pathways.

## 2 Model

### PROSSTT generates simulated scRNA-seq datasets in four steps

**Fig. 1.**
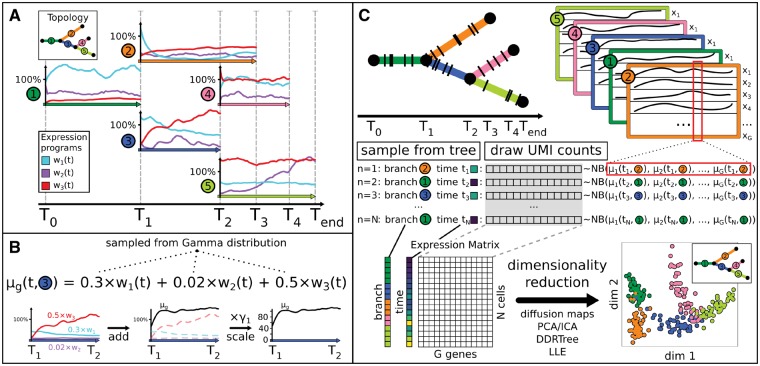
PROSSTT models the single-cell RNA-seq transcriptomes of cells differentiating along a (user-supplied or sampled) lineage tree. (**A**) A small number of gene expression programs is simulated by random walk along each of the tree branches (number of steps = integer branch length). Here, a double bifurcation is regulated by three expression programs. (**B**) Relative expected gene expression $\mu_{g}(t,b)$ is computed as weighted sum of the expression programs with randomly sampled weights (here: gene *g* in branch 3). Expected expression values are obtained by multiplying with a gene-dependent sampled scaling factor. (**C**) Cells are sampled from the tree as pairs of pseudotime *t* and branch *b*. For each pair, the corresponding average gene expression is retrieved and UMI counts sampled using a negative binomial distribution. Low-dimensional representations of the resulting gene expression matrix are similar to those of real data (Supplementary Section S1) and capture the lineage tree topology [diffusion map created with destiny (Angerer *et al.*, 2016)]

**1. Generate tree:** The topology of the lineage tree (number of branches, connectivity) and the length of each branch are read in or, alternatively, sampled. The integer branch lengths give the number of steps of the random walk (see next point) and correspond to the pseudotime duration [Fig. 1A (inset)]. The topology can also be linear.

**2. Simulate average gene expression along tree:** Gene expression levels are linear mixtures of a small number *K* (default: scales with number of bifurcations) functional expression programs *w_k_*. For each tree segment, we simulate the time evolution of expression programs by random walks with momentum term (see Fig. 1A and Supplementary Material). The mean expression of gene *g* in tree branch *b* at pseudotime *t* is a weighted sum of the *K* different programs *k*: $\mu_{g}(t,b)=\sum_{k=1}^{K}w_{k}(t,b)h_{k,g}$ (Fig. 1B). The weights $h_{k,g}$ are drawn from a gamma distribution (Supplementary Section S2.2).

**3. Sample cells from tree:** We offer multiple ways of sampling cells from a lineage tree: (i) sampling cells homogeneously along the tree, (ii) sampling centered diffusely around selected tree points, (iii) sampling with user-supplied density and (iv) specifying the velocity with which the process progresses and sampling the resulting density. (Fig. 1C left, Supplementary Section S2.3).

**4. Simulate UMI counts:** We simulate unique molecular identifier (UMI) counts using a negative binomial distribution. First, a scaling factor *s_n_* for the library size is drawn randomly for each cell *n* (see Supplementary Section S2.4). Following Grün *et al.* (2014) and Harris *et al.* (2018), we make the variance $\sigma_{g}^{2}$ depend on the expected expression $s_{n}\mu_{g}$ as $\sigma_{\mathrm{ng}}^{2}=\alpha_{g}{\left(s_{n}\mu_{g}\right)}^{2}+\beta_{g}(s_{n}\mu_{g})$. If $x_{n}(t,b)=(x_{1},x_{2},\ldots ,x_{G})$ is a cell at pseudotime *t* and branch *b*, the transcript counts are $x_{\mathrm{ng}}(t,b)∼\text{NegBin}\left(s_{n}\mu_{g}(t,b),\sigma_{\mathrm{ng}}^{2}(t,b)\right)$ (Fig. 1C, right). For each of *N* cells and each of *G* genes we draw the number of UMIs from the negative binomial, resulting in an *N *×* G* expression matrix, which can serve as input for tree inference algorithms.

Users can specify the topology of the lineage tree (any connected acyclic graph is acceptable), assign branch pseudotime lengths, adjust parameters for the gene expression programs and control the noise levels in the data. Default parameter values for $\alpha_{g},\beta_{g}$ and the base gene expression values were set in the range of parameters of real datasets (Supplementary Section S3). If provided with a real dataset, PROSSTT can learn hyperparameters that will generate simulated data with similar summary statistics.

## 3 Application

We generated 10 sets of 100 simulations each, for different degrees of topology complexity (from 1 up to 10 bifurcations). In another study, we used this dataset to assess the performance of our tool MERLoT and other methods (Parra *et al.*, 2018). We provide scripts with implementations of appropriate quality measures as well as the pipeline to generate the simulations and evaluate predictions by state-of-the-art software.

PROSSTT is capable of producing simulations with the summary statistics of true datasets, and can reproduce data faithfully in cases where the underlying lineage tree is available.

## 4 Conclusions

PROSSTT simulates scRNA-seq data for complex differentiation processes. Low-dimensional visualizations produced by tree reconstruction tools resemble those of real datasets. Increasingly complex datasets with uncertain biological ground truth are becoming available. PROSSTT can help the development of methods that can reconstruct such complex trees by facilitating their quantitative assessment. Furthermore, the modular nature of the software allows for easy extensions, for example PROSSTT could serve to test the influence of noise models and give biological insights into how to model and interpret scRNA-seq data.

## Funding

RGP is a long term EMBO postdoctoral fellow (ALTF 212-2016).

*Conflict of Interest*: none declared.

## Supplementary Material

btz078_Supplementary_Material
