# Supplementary material for: PROSSTT: probabilistic simulation of single-cell RNA-seq data for complex differentiation processes
Source: Bioinformatics. 2019 Feb 1;35(18):3517–9. doi: 10.1093/bioinformatics/btz078 (PMC6748774; doi:10.1093/bioinformatics/btz078)
Supplement: btz078_Supplementary_Material [file btz078_supplementary_material.pdf]

Supplementary Material for *PROSSTT: probabilistic simulation of single-cell RNA-seq data for complex differentiation processes*

Nikolaos Papadopoulos, R. Gonzalo Parra, Johannes Söding

Quantitative and Computational Biology Group, Max Planck Institute for Biophysical Chemistry, Göttingen, 37077, Germany

Detailed documentation for PROSSTT is available at `https://prosstt.readthedocs.io/en/latest/`. Multiple examples are available in the form of Jupyter notebooks in the `examples/` folder of the git repository: `https://github.com/soedinglab/prosstt/blob/master/examples`.

1 Diffusion maps of real and simulated datesets (Fig. S1)

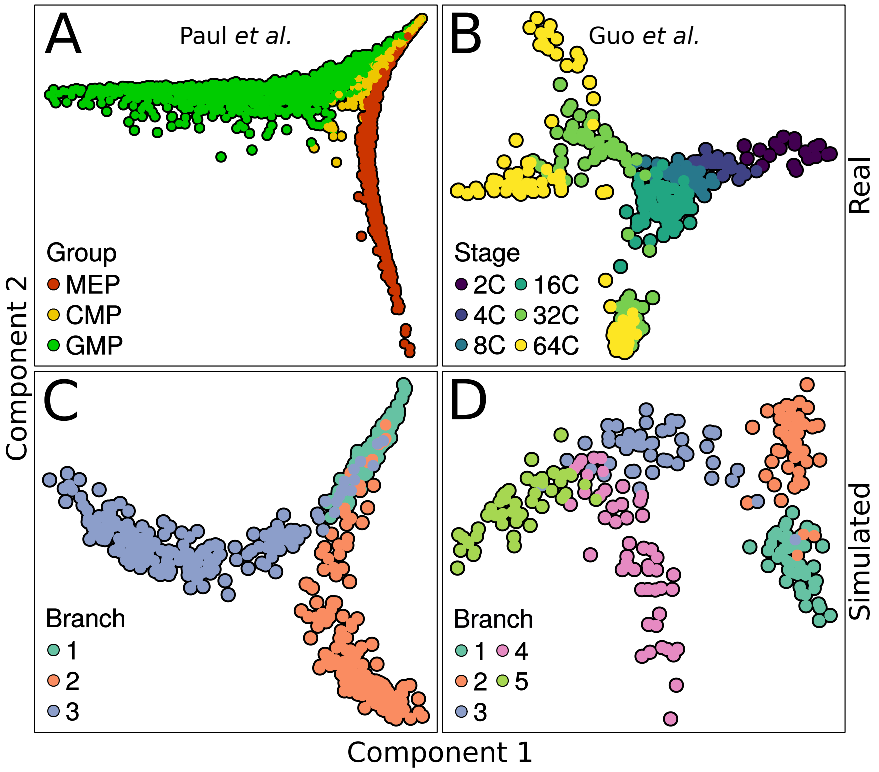

Figure S1: Diffusion maps of real (left) and simulated (right) datasets.

A: Mouse bone marrow cells were profiled using scRNA-seq (Paul *et al.*, 2015). An expression matrix containing 2730 cells and 3459 genes was obtained, and the cells were grouped in 19 transcriptionally homogenous clusters, as described by the authors. Using the expression of known marker genes, the cells were assigned to three broad clusters - megakaryocyte/erythroid progenitors, common myeloid progenitors, and granulocyte/macrophage progenitors. A diffusion map with default parameters was calculated with *destiny*, and the first two diffusion components were used to plot the figure.

B: Mice embryos up to and including the 64 cell stage were profiled using qRT-PCR (Guo *et al.*, 2010). An expression matrix containing 428 cells and 48 genes was obtained. The embryonal stage of each cell is known. The protocol described in the *destiny* vignette for data normalization was used in order to derive the final gene expression matrix. A diffusion map with global *sigma* option was calculated with *destiny*. To simplify visualization, the third diffusion component was rotated by 30 degrees onto the first. The transformed 1st-3rd diffusion component and the 2nd were used to plot the figure.

C: scRNA-seq data of a differentiation process with a single bifurcation, simulated using PROSSTT. This particular simulated data belongs to the simulations produced for the benchmark of the tool “MERLoT” (single bifurcation, simulation #38, Parra *et al.* (2018)). It contains 758 genes, simulated using 10 functional components. Each branch was 50 pseudotime

units long. The variance parameters for the negative binomials were sampled from $\alpha_g \sim e^x, x \in N(\log(0.2), 0.3)$ and $\beta_g \sim e^x, x \in N(\log(2), 0.3)$ respectively. The simulation for MERLoT originally contained 150 cells, but to improve visualization, the average gene expression trajectories from simulation #38 (simulation `benchmark1/test38/` in `http://wwwuser.gwdg.de/~compbiol/merlot/`) were reused to sample the whole lineage tree 4-fold, resulting in 600 cells. The first and second diffusion components were used for visualization.

D: scRNA-seq data of a differentiation process with a double bifurcation, simulated using PROSSTT. This particular simulated data belongs to the simulations produced for the benchmark of the tool "MERLoT" (double bifurcation, simulation `benchmark1/test24/` in `http://wwwuser.gwdg.de/~compbiol/merlot/`). It contains 250 cells and 836 genes, simulated using 10 functional components. Each branch is 50 pseudotime units long, and their connectivity (lineage tree topology) is described by $[[0, 1], [0, 2], [2, 3], [2, 4]]$, where branch 0 is the branch with pseudotime 0, where the differentiation starts. The variance parameters for the negative binomials were sampled from $\alpha_g \sim e^x, x \in N(\log(0.2), 0.3)$ and $\beta_g \sim e^x, x \in N(\log(2), 0.3)$ respectively.

The R script as well as the data that were used to produce the figures can be found in the `https://github.com/soedinglab/prosstt-scripts/` repository.

2 PROSSTT simulation structure

A typical PROSSTT simulation is performed in the following steps, which will be explained in detail below.

0. Initialize tree:
   - 0a. Set number of expression programs K.
   - 0b. Set number of genes G.
1. Generate tree:
   - 1a. Set lineage tree topology.
   - 1b. Set length of each branch in pseudotime units.
2. Simulate average gene expression along tree.
   - 2a. Simulate expression programs for each branch.
   - 2b. Randomly assign programs to genes.
   - 2c. Calculate relative average gene expression change.
   - 2d. Simulate base gene expression values.
   - 2e. Scale relative average gene expression change.
3. Sample cells from lineage tree according to a sampling strategy.
4. Sample variance parameters and simulate UMI counts for each gene g and cell n.

2.1 Generate tree

The various options available to initialize a tree are documented in `https://prosstt.readthedocs.io/en/latest/step1.html`. PROSSTT can simulate topologies in the form of connected acyclic graphs (such as linear differentiations or binary trees). Additionally, PROSSTT allows the simulation of committed lineages that separate later - two branches that will only diverge away from each other towards the end of their shared length.

2.2 Simulate average gene expression along the tree

2.2.a Simulate expression programs for each branch

In PROSSTT, gene expression is controlled by a small number of expression programs $K \ll G$. An expression program k encodes impulses for change in relative mean expression during pseudotime on a linear segment of the lineage tree (branch b). Intuitively, expression programs can be described as coordinated fold-change in average gene expression caused by any cellular process that has a time component.

For example, activation of a group of transcription factors A will upregulate some genes (expression increase over time) while transcription factor group B will cause downregulation (expression decrease over time) of some of the same genes. The influence of each factor is one expression program ("steady expression for 5 pseudotime units (PUs) and upregulation by 50% over 10 PUs", "downregulation by 30% over 15 PUs"). The fold-change in the expression of each gene will be an interplay

of the different influences (for a gene that is equally influenced by A and B it would be "downregulation by 30% over 5 PUs and upregulation by 20% over 10 PUs").

We simulate this change in every segment of the lineage tree with a random walk with a momentum term η, an amortized diffusion process that starts in $[0, 1.5]$ and moves freely. This diffusion takes place in log-space and is presented in Algorithm 1. For each program k in each branch b, $W_{b,k}[t]$ describes the location of the diffusing particle in time t, while $V_{b,k}[t]$ describes its velocity. The position of the particle at the time step $t+1$ is determined by its current position and its current velocity. In the following, let T_b be the length of the branch in pseudotime units, $\mathcal{N}(\mu, \sigma^2)$ a normal distribution with mean μ and variance σ^2 and $\mathcal{U}(0, 1.5)$ the uniform distribution between 0 and 1.5.

$W_{b,k}[0] = 0$;
$V_{b,k}[0] \sim \mathcal{N}(0, 0.2)$;
$\eta \sim \mathcal{U}(0, 1.5)$;
while $t \leq T_b$ **do**
  $W_{b,k}[t+1] \leftarrow W_{b,k}[t] + V_{b,k}[t]$;
  $\epsilon_t \sim N(0, 2/T_b)$;
  $V_{b,k}[t+1] \leftarrow 0.95 V_{b,k}[t] + \epsilon_t + \eta V_{b,k}[t]$;
  $t \leftarrow t+1$
end

Algorithm 1: Amortized diffusion in log-space

In each new time step $t+1$ the location $W_{b,k}[t+1]$ is updated by moving away from point $W_{b,k}[t]$ with speed $V_{b,k}[t]$. Then, the speed at the next step is updated: It is going to be almost the speed from the previous step ($0.95 V_{b,k}[t]$) plus a small random change ϵ.

The overall change in position $\sum_{t=1}^{T_b} \epsilon_t$ is a sum of independent, identically distributed changes $\epsilon_t \sim \mathcal{N}(0, 1/T_b)$, and so is itself normally distributed with mean 0 and variance equal to the sum of the ϵ_t's.

$$\sigma_{total}^2 = \sum_{t=1}^{T_b} \sigma_{\epsilon_t}^2 = T_b \sigma_{\epsilon_t}^2 = T_b \frac{1}{T_b} = 1 \tag{1}$$

This means that the expression fold change inside a linear segment is independent of the branch length in pseudotime units.

The diffusions of all K programs for branch b of the lineage tree can be summarized in a matrix $\mathbf{W}_b \in K \times T_b$, where each row is the random walk that represents program k in branch b as a function of pseudotime T_b ($W_{b,k}$). This process is repeated for all tree branches.

The number K of expression programs depends on the complexity of the simulated process. This is intuitively clear: one expression program would be enough to cause a single bifurcation, e.g. by upregulating all genes it influences in branch A and downregulating them in branch B. For two bifurcations, one expression program is not enough: if we apply the same "trick" at the end of branch A in order to create branches C (further upregulation) and D (downregulation), then the cells in branch D will be indistinguishable from the cells in branch A. A more nuanced approach is needed. This is further complicated by the fact that expression programs are random walks, and as such one cannot guarantee that each and every one of them will behave in a sufficiently different manner in parallel branches. PROSSTT therefore scales the number of expression programs quasi proprotionally to the number of branch points P present in the topology:

$$K = 5 \cdot P + x,\ x \sim \tilde{\mathcal{U}}(3, 20) \tag{2}$$

where $\tilde{\mathcal{U}}(3, 20)$ is the discrete uniform distribution between 3 and 20.

To alleviate the situation a bit more, PROSSTT imposes a correlation restriction on expression programs. Programs in the same branch are not allowed to have a Pearson's correlation coefficient above a certain threshold (default value 0.2). Due to the absence of redundancy, a small number of expression programs and their combinations are enough to simulate all the transcriptional variability.

2.2.b Randomly assign genes to programs

The relative average expression of gene g in tree branch b at pseudotime t is, by default, a weighted sum of the K different programs: $\mu_g(t, b) = \sum_{k=1}^{K} w_k(t, b) h_{k,g}$, where $h_{k,g} \sim \Gamma(\alpha)$. The coefficients can be summed up in a matrix $\mathbf{H} \in K \times G$. The shape α is set to the minimum of $1/K$ and 0.05. This reflects the fact that the number of expression programs grows with the topology complexity. If α had a static value, then simulations with more expression programs genes would have a higher average relative expression, leading to uneven simulations.

Alternatively, expression program contribution can also be set by choosing two expression programs to influence each gene. Here the coefficients are sampled from a beta distribution $B(\alpha, \beta)$ (default values: $\alpha = 2, \beta = 2$), while the contribution of all other expression programs is set to zero. Obviously, no additional balancing is required in this case.

2.2.c Calculate relative average gene expression

Multiplication of the coefficient matrix to the expression programs creates matrices that contain relative gene expression over pseudotime: $\mathbf{W}_b^T \times \mathbf{H} = \mathbf{M'}_b$ where $\mathbf{M'}_b \in T_b \times G$.

If expression programs on parallel branches (branches that are children of the same parent branch) correlate too much, then average gene expression will be very similar on both branches, with the result that they will not be easily distinguished. PROSSTT can resample the expression programs for the parallel branches until a certain proportion of genes is anticorrelated (Pearson correlation coefficient below 0). This is turned off by default.

Occasionally, some genes will have an extreme fold change, over a particular threshold (default: $e^8 \approx 3000$). If this happens, PROSSTT will re-simulate the offending branches to ensure that the expression of one gene does not dominate the dimensionality reduction of the simulated dataset.

2.2.d Simulate base gene expression

In order to translate fold-change in gene expression to change in absolute average gene expression over pseudotime, the relative expression encoded in $\mathbf{M'}_b$ must be scaled appropriately. This is done by sampling a base expression value for each gene. Intuitively, this is the expression of gene g in an unperturbed system; in the course of differentiation, differential expression of gene g happens respective to this base expression value.

Base expression values are sampled as $e^x, x \sim \mathcal{N}(0.8, 1)$. If a gene has an extreme fold-expression change over the lineage tree, then PROSSTT will try to pick a base expression value such that the absolute mean expression of the gene does not exceed a certain threshold value (default: 5000).

2.2.e Scale relative average gene expression change

This is a multiplication of the base expression values for each gene (vector $\hat{\mathbf{b}}$ of size G) with the relative expression matrix for each branch: $\mathbf{M}_b = \mathbf{M'}_b \cdot \hat{\mathbf{b}}$, where $\mathbf{M}_b \in T_b \times G$ is a matrix that contains absolute gene expression over time for branch b.

2.3 Sampling strategies

"Sampling" is the task of picking cells at the different pseudotime points along the lineage tree structure to simulate their expression. Formally, it consists of picking combinations of pseudotime points and branches (t, b). PROSSTT includes three sampling strategies. The modular nature of the program facilitates the inclusion of user-customized sampling strategies. Jupyter notebooks with example code for all cases are provided at `https://github.com/soedinglab/prosstt/tree/master/examples`.

2.3.a Sampling the whole lineage tree

Every point on the lineage tree is sampled n times. This mode produces simulations that are the easiest for trajectory inference algorithms, since it covers all possible instances of the model homogeneously. A variant of this mode will sample all pseudotime points n times, but will pick a branch randomly if multiple branches are available at the given pseudotime point. This mode still produces relatively easy simulations, with only a few small gaps that the algorithms must impute.

2.3.b Sampling a pseudotime series experiment

This option creates a simulation that resembles real data the most. scRNA-seq is usually performed as a time series experiment, where groups of cells are taken out of a culture or tissue at different time points $t'_1, t'_2, ..., t'_s$. In all snapshots, cells at the same sample time t'_i are going to be distributed around the same underlying differentiation time. In our model, given the amounts of cells sampled at each sample pseudotime point t'_i, we simulate cells with pseudotime values distributed around it. If multiple branches are possible, one is assigned randomly. This strategy produces the hardest simulations, as the gaps the algorithms must fill are going to be larger, and the density of cells along pseudotime is not uniform. For an example, refer to `https://github.com/soedinglab/prosstt/blob/master/examples/sample_pseudotime_series.ipynb`.

2.3.c Density sampling

Users might want to specify a sampling density to mimic relative cell abundance at different timepoints (e.g. a differentiation where most cells have already reached a cell fate but some linger in previous stages). Unless otherwise specified, the density of a simulated tree is uniform, i.e. all tree points are equally likely to be sampled. Consider `https://github.com/soedinglab/prosstt/blob/master/examples/density_sampling.ipynb` for an example of density sampling.

2.3.d Cell velocity

A different reason why parts of the lineage tree would be more or less dense is the speed of differentiation - cells might need a long time to make a cell fate decision (bottleneck) but differentiate quickly once that point has been passed. Users can specify differentiation velocities, which are translated to density behind the scenes. For a demonstration, refer to `https://github.com/soedinglab/prosstt/blob/master/examples/velocity.ipynb`.

Density sampling and pseudotime series can be combined to produce datasets with more realistic cell abundances. For an example, see `https://github.com/soedinglab/prosstt/blob/master/examples/combined_sampling.ipynb`.

2.4 Sampling variance hyperparameters and simulating UMI counts

We simulate unique molecular identifier (UMI) counts using a negative binomial distribution. First, a scaling factor s_n for the library size is drawn randomly for each cell n. The variance σ_g^2 depends on the average gene expression $s_n\mu_g$ as $\sigma_{ng}^2 = \alpha_g(s_n\mu_g)^2 + \beta_g(s_n\mu_g)$. For every cell n, sampled in time t and branch b, and each gene g, the transcript counts are $x_{ng}(t,b) \sim \text{NegBin}\left(s_n\mu_g(t,b), \sigma_{ng}^2(t,b)\right)$.

The hyperparameters are drawn from exponentiated normal distributions. Specifically:

- α_g is drawn from $e^x, x \in \mathcal{N}(\log(0.4), \log(2))$
- β_g is drawn from $e^x, x \in \mathcal{N}(\log(2), \log(2))$
- s_n is drawn from $e^x, x \in \mathcal{N}(\log(1), \log(0.7))$

3 Modelling count data from UMI protocols: motivating model and parameter choices

Grün *et al.* (2014) proposed that the negative binomial distribution, a discrete probability distribution that can model variance independently of the mean, provides a robust model for transcript count data, and supported their statement with analysis of a UMI dataset. We validated this proposition by fitting log-normal, Poisson, and negative binomial distributions on transcript count data from four recent publications (Grün *et al.*, 2014; Hashimshony *et al.*, 2016; Islam *et al.*, 2014; Zeisel *et al.*, 2015). In the first three a population of cells of the same type are analyzed, while Zeisel *et al.* (2015) find 9 major and 47 minor subtypes of cells in the mouse cortex and hippocampus. Cells of the same type (or subtype) can reasonably be expected to have similar expression values, and therefore we can use them to analyze various properties of the data. This included a total of 139966 genes in 3119 cells and 53 subgroups. It is important that no differentiating cells are included in these datasets, as average gene expression changes during differentiation, considerably complicating analysis of count data distribution. A detailed overview of these datasets can be found in Table S1.

The transcript count data was normalized per cell according to library size before fitting:

$$x_{ng} = \frac{x_{ng}}{S_n} \frac{\sum_{n=1}^{N} S_n}{N} \tag{3}$$

where $S_n = \sum_{g=1}^{G} x_{ng}$.

For each gene in each of the 53 subgroups, we calculated the Bayes Information Criterion for the negative binomial, Poisson and log-normal fits, according to eq. 4.139 in Bishop (2006). When considering the genes where one of the three models has positive or strong evidence in its favor (BIC difference of 2 or higher), the negative binomial distributions explain the transcript count distribution of a larger number of genes in most subgroups (see `prosstt-examples/plots.ipynb` notebook).

We used the log-normal implementation of the python *scipy* package (`scipy.stats.lognorm`). For the Poisson and negative binomial distributions, we implemented probability density functions and their derivatives and fitted them using maximum likelihood and the python `scipy.optimize` package with its implementation of the L-BFGS-B algorithm (Zhu *et al.*, 1997). For the negative binomial distributions we used the p, r parametrization and a formulation that can deal with fractional counts (after correcting for library size):

$$\Pr(X = k) = \frac{\Gamma(r+k)}{\Gamma(k+1)\,\Gamma(r)} p^k (1-p)^r \quad \text{for } k \in \mathbb{R} \tag{4}$$

In general, for PROSSTT, we assume a variance model where the variance of the expression of a gene depends on the square of the mean in the form $\sigma_g^2 = \alpha_g\mu_g^2 + \beta_g\mu_g$. This is the simplest negative binomial model that accounts for the overdispersion observed by Grün and others, while still allowing genes to have a Poisson expression pattern ($\alpha_g \simeq 0$ and $\beta_g \simeq 1$).

In order to fit α, β values from the data we need a dataset where the average gene expression values differ between groups of cells. This condition was only satisfied for the Zeisel dataset, which was therefore used. Fitting negative binomials generated parameters $p_{k,g}, r_{k,g}$ for each gene g in each cell cluster k. We used these values to calculate the mean $\mu_{k,g}$ and

variance $\sigma^2_{k,g}$ of the count distribution of each gene in each group. We then minimized the Euclidean distance between the calculated and postulated variance over all K clusters of cells:

$$f(\alpha_g, \beta_g | \mu_{k,g}, \sigma_{k,g}) = \sqrt{\sum_{k=1}^{K} \sigma^2_{k,g} - (\alpha_g \mu^2_{k,g} + \beta_g \mu_{k,g})} \tag{5}$$

The partial derivatives are (leaving out the g index and the data):

$$\frac{\partial f}{\partial \alpha} = \frac{-\sum_{k=1}^{K} \mu_k^2 (\sigma_k^2 - \alpha \mu_k^2 - \beta \mu_k)}{f(\alpha, \beta)} \tag{6}$$

$$\frac{\partial f}{\partial \beta} = \frac{-\sum_{k=1}^{K} \mu_k (\sigma_k^2 - \alpha \mu_k^2 - \beta \mu_k)}{f(\alpha, \beta)} \tag{7}$$

We conclude that it is appropriate to model the distribution of transcript count data for gene g with:

$$\mathbf{x}_g \sim \text{NegBin}(\mu_g, \sigma_g^2 = \alpha_g \mu_g^2 + \beta \mu_g) \tag{8}$$

These fits can be used to study appropriate ranges and distributions for the relevant hyperparameters of the negative binomial distributions. We fit log-normal distributions for α_g, β_g, cell library size, and average gene expression. The results are summarized in Table S1.

parameter	data average	PROSSTT default value
α mean	0.4187	0.4
α variance	6.1448	2
β mean	1.8375	2
β variance	1.9401	2
libr. size mean	0.9034	1
libr. size variance	1.5577	0.7
avg. gene expr. mean	0.9309	0.8
avg. gene expr. variance	1.7747	1

Table S1: Average parameter values from the fits and correponding default values in PROSSTT.

The default parameters of PROSSTT are picked very close to the average of the fitted parameter values, with the exception of average gene expression. This is because of how PROSSTT models gene expression; the values listed as PROSSTT defaults here are the values for base gene expression. Before PROSSTT uses average gene expression, it is scaled by its fold-change, resulting in a much broader distribution.

Detailed analysis, plots, as well as the code can be found online, in `https://github.com/soedinglab/prosstt-scripts/`. A Jupyter notebook for each of the four datasets contains data processing, normalization and fitting. Another notebook (`plot.ipynb`) contains plots and summaries of the results.

The choice of variance hyperparameters is of critical importance, as high variance will obscure the underlying structure of the data and make reconstruction of the lineage tree impossible (for example `https://github.com/soedinglab/prosstt/blob/master/examples/variance_sim.ipynb`).

4 Prediction evaluation and visualization

Lineage tree inference algorithms must succeed at two tasks: a) to place biologically similar cells close to each other and biologically dissimilar cells away from each other, and b) to place cells in such a way that their distance from the root of the lineage tree (in some measure) reflects their progress in the developmental pathway. The success of each task depends, to a degree, on the success of the other. To the best of our knowledge, there is no single measure that can encompass both at once, so we assess each separately.

4.1 Grouping similar cells

The first task can be interpreted in multiple ways. For example, since PROSSTT provides pseudotime labels for all cells, one can define two cells as "similar" if they are below a certain pseudotime threshold. Evaluation would consist of determining the nearest neighbours of each cell in the trajectory reconstruction and scoring the neighbourhood by its mean pseudotime distance from the cell. However, cell distances after dimensionality reduction depend on the difference in gene expression,

which is not proportional to the pseudotime. At branch points, for example, the average expression in one branch may change faster than on the other, leading to larger manifold distances between cells despite similar pseudotime distances.

Instead, one can use the lineage tree structure, and consider cells in the same segment (branch) sufficiently similar to each other. These can be compared to the branch assignments produced by a trajectory inference algorithm that reconstructs the lineage tree, reducing a) to a clustering problem. Given a cell c, let $Tr(c)$ be the cluster identity of c in the ground truth and $A(c)$ the cluster assigned to c by the algorithm. We define a pair of two cells c_i, c_j as a...

$$\begin{array}{lll} \text{true positive (TP)} & \text{if} & \mathrm{Tr}(c_i) = \mathrm{Tr}(c_j) \wedge \mathrm{A}(c_i) = \mathrm{A}(c_j) \\ \text{true negative (TN)} & \text{if} & \mathrm{Tr}(c_i) \neq \mathrm{Tr}(c_j) \wedge \mathrm{A}(c_i) \neq \mathrm{A}(c_j) \\ \text{false positive (FP)} & \text{if} & \mathrm{Tr}(c_i) \neq \mathrm{Tr}(c_j) \wedge \mathrm{A}(c_i) = \mathrm{A}(c_j) \\ \text{false negative (FN)} & \text{if} & \mathrm{Tr}(c_i) = \mathrm{Tr}(c_j) \wedge \mathrm{A}(c_i) \neq \mathrm{A}(c_j) \end{array}$$

Using these four values, many popular clustering indices can be computed: the F1 measure is $2PR/(P+R)$ and the Fowkles-Mallows index is $\sqrt{PR}$ (where P is the precision $TP/(TP+FP)$ and R is the recall $TP/(TP+FN)$). The Matthews Correlation Coefficient is

$$\frac{(TP \cdot TN - FP \cdot FN)}{\sqrt{((TP+FP)(TP+FN)(TN+FP)(TN+FN))}},$$

and the Jaccard Index is $TP/(TP+FP+FN)$, while the (unweighted) Rand index is $(TP+TN)/(N)$, where N is the number of all possible pairs ($N = TP+FP+TN+FN$).

While all the aforementioned indices and measures are well established, they are suboptimal performance indicators for the problem at hand, since they don't take cluster structure into consideration. As the simulated lineage trees become bigger and more complex, the number of cell pairs that are not in the same tree segment is going to grow much faster than the number of cell pairs in different tree segments. The number of TPs will grow much slower than the number of TNs, something that will, for example, inflate the the MCC index. Additionally, the number of possible FNs becomes much higher with each additional segment added to the tree, something that affects the Jaccard index and the F1 score. In short, these measures don't take into account the number of clusters in the data, and as such are suboptimal descriptors of clustering performance in comparison to NMI.

In our opinion the NMI is best for assessment of branch assignment, since it captures the amount of information present in the original clustering that was recovered by the prediction (values between 0 and 1). It corrects the effect of agreement solely due to chance between clusterings by using a hypergeometric background distribution and punishes overbranching and merging branches almost equally (Vinh *et al.*, 2010). Given the predicted and real cluster assignments U and V, NMI is defined as

$$\mathrm{NMI}(U,V) = \frac{\mathrm{MI}(U,V) - \mathbb{E}[\mathrm{MI}(U,V)]}{\max\{H(U),H(V)\} - \mathbb{E}[\mathrm{MI}(U,V)]}.$$

4.2 Evaluating cell ordering

Evaluating the performance of the different methods consists of quantifying the degree of agreement between the true/labeled and the predicted orderings provided by the different algorithms. Pseudotime only establishes a partial ordering on cells and no absolute time. Therefore, cells on branches not passed through one after the other cannot be compared. We find the longest path in the tree (from the root to a leaf) and compare the predicted pseudotime with the simulated one for the cells on this path. We used the Goodman-Kruskall index and the Kendall index (weighted and unweighted) to assess pseudotime prediction, as proposed by Campello and Hruschka (2009). All four indices count how many pairs of cells have been ordered correctly (S_+) or incorrectly (S_-) and produce similar results for the benchmark. The unweighted Goodman-Kruskall index is the simplest approach: $(S_+ - S_-)/(S_+ + S_-)$.

5 Divergence Analysis

We generated benchmark datasets for MERLoT (Parra *et al.*, 2018) with PROSSTT and Splatter (Zappia *et al.*, 2017). Multiple methods did not perform according to expectations. The issue was particularly obvious in the evaluation of pseudotime prediction, where Monocle2 (Trapnell *et al.*, 2014) sank to the level of random predictions, and MERLoT, which, even though it excelled at branch assignment, dropped off to quite low accuracy for large numbers of bifurcations. Additionally, TSCAN (Ji and Ji, 2016), which in the PROSSTT benchmark proved to be competent in branch assignment, returned completely nonsensical predictions for data simulated by Splatter.

As these methods have all been applied successfully on real data, we chose to examine the simulations. By visual inspection of the manifold embeddings we observed that the manifold embeddings of Splatter simulations often presented "short-circuits", where parts of the lineage tree seemed to fold back to preceding tree segments, effectively creating cycles.

While branches were often separated correctly (i.e. the cell clustering was correct), they were connected in wrong ways, decreasing the pseudotime prediction accuracy.

Since the manifold embeddings are a projection that aims to retain the most important dynamics in the data, we hypothesized that the reason for these short-circuits was that the offending branches did not diverge enough, or even converged towards previous tree segments. In terms of gene expression, this means that differentiating phenotypes (captured transcriptomes) were either not different enough or that they converged towards preceding phenotypes.

To test this hypothesis, we measured the distance of each waypoint (endpoint or branchpoint) from the origin, and normalized it by the average branch length in the path that led to it. The calculations described below were performed on the simulated gene expression data, after normalization for library size and log-transformation (see scripts `divergence_euclidean.R` and `divergence_diffmaps.R` in `https://github.com/soedinglab/merlot-scripts`).

As explained in Fig. S2, we retrieved the cells with minimum and maximum pseudotime in each branch (start cells and end cells respectively), calculated their pairwise distances, and defined their average d_b as the length of branch b. Next, we took the cells with globally minimum pseudotime and calculated their distances from all end cells. This is the minimum direct distance d'_b of each branch b from the origin. We normalized each d'_b with $\bar{d}_b = \frac{1}{|p|}\sum_{b' \in p} d_{b'}$, where p is the path $[b_0, b_1, ...b]$ from the origin branch b_0 to branch b. Effectively, this yields the distance from the origin measured in average branch lengths. Pooling the normalized distances from all paths of equal length (especially since all branches have the same length in the PROSSTT and Splatter simulations) shows how much the change in expression values correlates with pseudotime.

The divergence curve of PROSSTT (Fig. S3A) shows what we expected: monotonic growth (i.e. longer paths are on average further away from the origin) and values above 1, indicating that paths with two branches or more consistently end outside a one-branch-length radius from the origin, something that reduces the possibility of wrong assignments from the methods.

On the contrary, the divergence curve of Splatter (Fig. S3B) stays almost completely below 1 and even shows a slightly negative slope. This means that expression profiles of cells in later differentiation stages dont move further from the origin with increasing pseudotime length. We believe this happens because Splatter does not include co-regulation in its differentiation simulation model. This leads to the differences between branches being completely random, and while this may work to separate two diverging branches from each other, it does not seem to yield realistic diverging cell lineage trees.

As a control, we performed the divergence analysis in the diffusion maps created by destiny (Angerer *et al.*, 2016) for the benchmark (panels G,H in Fig. S3). Diffusion distance was proposed as a measure of cell similarity in the original paper Haghverdi *et al.* (2014), and was the most effective of the embeddings used in this study. We see the same trends as in gene expression space; the divergence curve of PROSSTT has positive slope and is consistently above 1, while the Splatter curve has a (clear) negative slope and stays below 1.

After quantifying divergence in both PROSSTT and Splatter simulations, we conclude that the simulations produced by Splatter have inherent characteristics that prevent algorithms that try to find a global structure (like MERLoT and monocle) from reaching their full potential. Consequently, we decided to only use simpler topologies for the Splatter benchmark (up to 4 bifurcations), where the impact of short-circuits was less dominant.

6 Learning from real data

While PROSSTT's default parameters are sampled from realistic ranges, users may want to simulate data with characteristics similar to real datasets, or even reproduce a real dataset. PROSSTT can learn hyperparameters and average gene expression from a real dataset, and produce simulations with similar summary statistics. This is demonstrated via Jupyter notebooks (in `https://github.com/soedinglab/prosstt/tree/master/examples` or the examples directory of the PROSSTT repository) for four different datasets, produced with different experimental protocols and from vastly different systems:

- Axolotl limb regeneration (10x Genomics) (Gerber *et al.*, 2018) `compare_axolotl.ipynb`
- Developing whole hydra (Drop-seq) (Siebert *et al.*, 2018) `compare_hydra.ipynb`
- Hematopoietic stem cells (index-omics) (Velten *et al.*, 2017) `compare_velten.ipynb`
- Multiple timepoints whole zebrafish embrya (Drop-seq) (Farrell *et al.*, 2018) `compare_zebrafish.ipynb`

In the case where a lineage tree has been reconstructed, PROSSTT can be configured to reproduce the process. We demonstrate an example (`https://github.com/soedinglab/prosstt/blob/master/examples/reproduce_axolotl.ipynb`) for axolotl limb regeneration where the lineage tree has been reconstructed with MERLoT (Parra *et al.*, 2018) on diffusion maps (Haghverdi *et al.*, 2014).

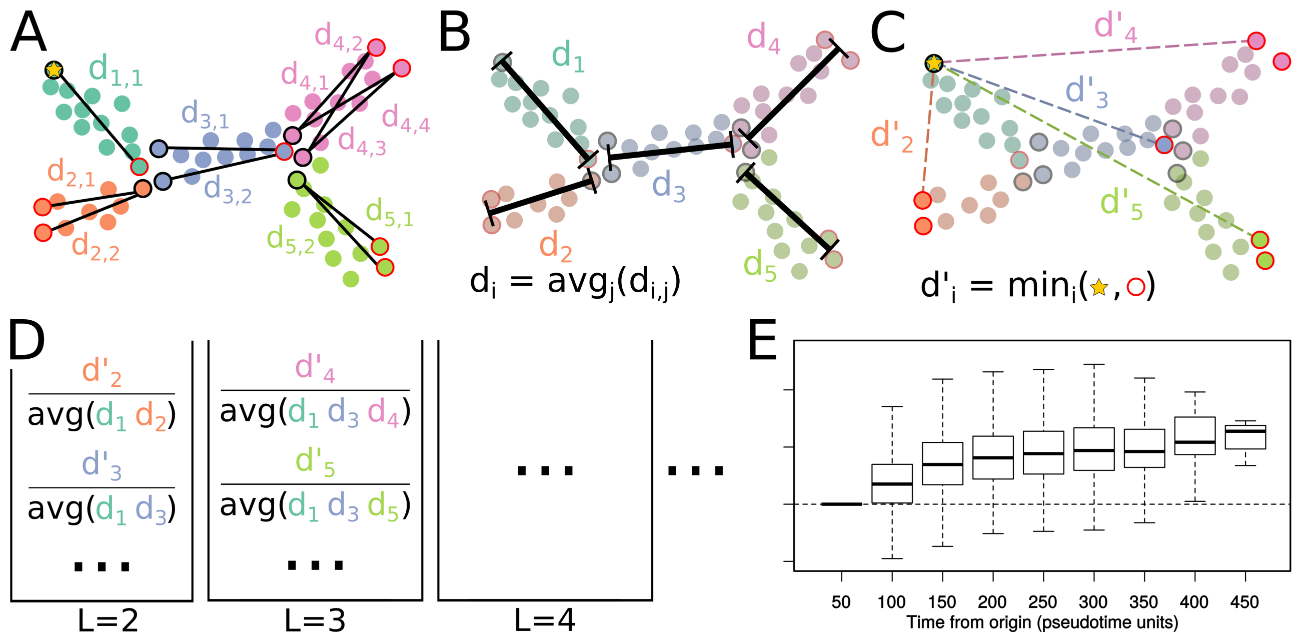

Figure S2: **Divergence analysis.** **(A)** We locate the cells with minimum and maximum pseudotime in each branch and calculate their pairwise distances. **(B)** The average of these distances is the branch length. **(C)** We calculate the minimum direct distance of the origin of the differentiation (minimum pseudotime of first branch) to the other waypoints (maximum pseudotime of each branch). **(D)** We take all on-tree paths from the origin to a waypoint and normalize the minimum direct distance via the average branch length in the path. After repeating steps (A)-(C) for all simulations in the set, we group these values by path length (in branches). **(E)** The boxplots of each path bin constitute the divergence curve of the simulations. If the branches all have the same pseudotime length, then distance to waypoints is equivalent to the pseudotime that has passed since the origin.

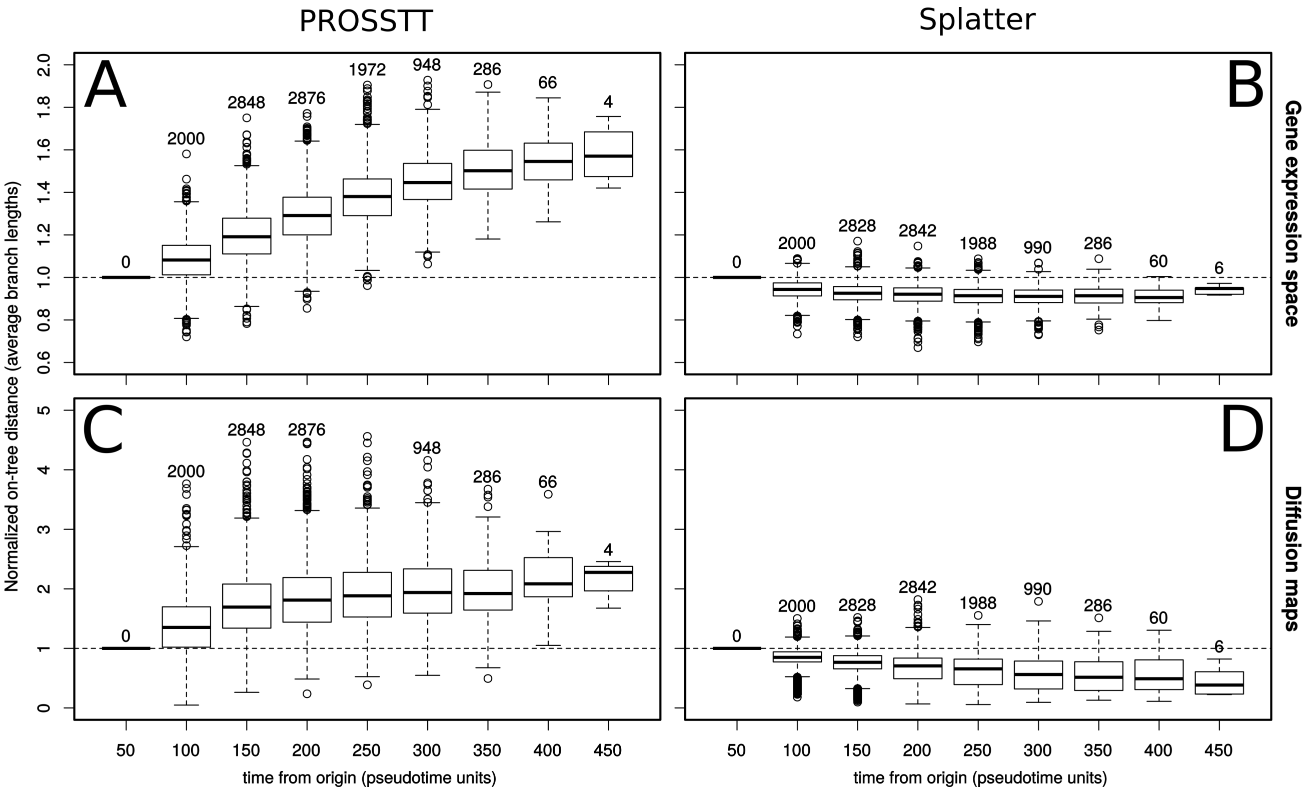

Figure S3: **Divergence analysis results.** Divergence analysis for PROSSTT (left) and Splatter (right) simulations in gene expression space (upper panels) or in diffusion space (lower panels).

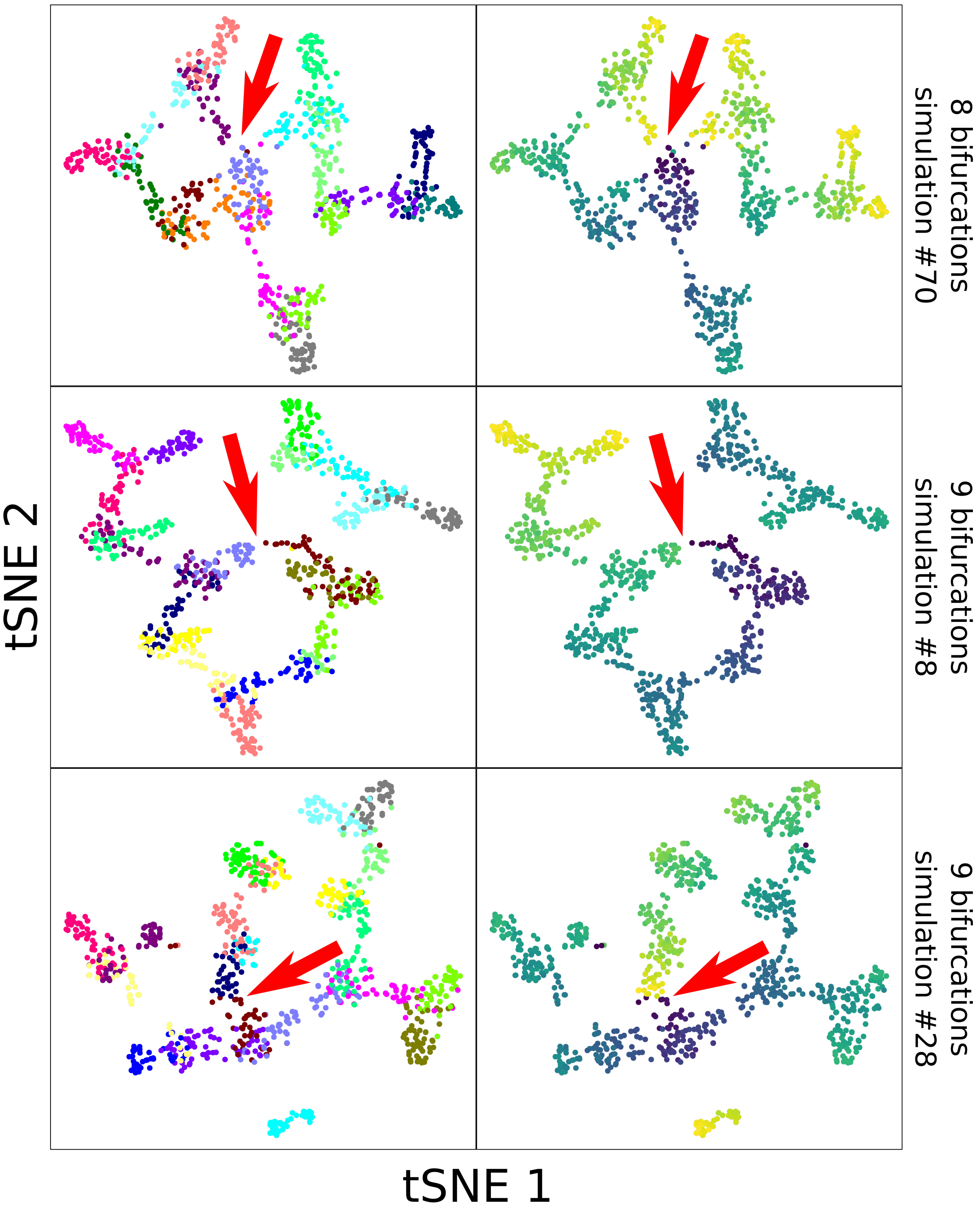

Figure S4: **Short circuits.** tSNE plots of Splatter simulations that show short-circuits. On the left side the colors denote the branches. On the right side the color denotes the pseudotime, from low (dark blue) to high (yellow). The red arrows point to the exact points where the short-circuit takes place. In all cases, cells with low and high pseudotime are so close to each other that the shape of the lineage tree is not clear, leading to problems in the tree reconstruction.

7 Programming and tools

Analysis and development was done in Python 3.6 (van Rossum, 1995) and R (R Core Team, 2017). IPython (Pérez and Granger, 2007) notebooks were used for the development of the simulation suite while Rstudio (RStudio Team, 2015) was used to write and analyze the evaluation code. PROSSTT was programmed in Python 3 using NumPy (van der Walt *et al.*, 2011) and SciPy (Jones *et al.*, 01). Plotting was performed with matplotlib (Hunter, 2007) and R base graphics. The `parallel` (Tange, 2011) bash utility was used to speed up simulations.

7.1 Implementation and availability

We provide code and scripts for the evaluation of trajectory inference predictions on data simulated by PROSSTT as the R package *prosstt*, available at `https://github.com/soedinglab/prosstt-r`. We have also implemented a plotting function that visualizes trajectory inference predictions on PROSSTT simulations.

Acknowledgements

The authors wish to think Tobias Gerber for providing the reconstructed tree object from the axolotl limb regeneration study (Gerber *et al.*, 2018).

References

Angerer, P., Haghverdi, L., Büttner, M., Theis, F. J., Marr, C., and Buettner, F. (2016). destiny: diffusion maps for large-scale single-cell data in r. *Bioinformatics*, **32**(8), 1241–1243.

Bishop, C. M. (2006). *Pattern Recognition and Machine Learning (Information Science and Statistics)*. Springer-Verlag New York, Inc., Secaucus, NJ, USA.

Campello, R. J. G. B. and Hruschka, E. R. (2009). On comparing two sequences of numbers and its applications to clustering analysis. *Inf. Sci.*, **179**(8), 1025–1039.

Farrell, J. A., Wang, Y., Riesenfeld, S. J., Shekhar, K., Regev, A., and Schier, A. F. (2018). Single-cell reconstruction of developmental trajectories during zebrafish embryogenesis. *Science*, **3131**(April), eaar3131.

Gerber, T., Murawala, P., Knapp, D., Masselink, W., Schuez, M., Hermann, S., Gac-Santel, M., Nowoshilow, S., Kageyama, J., Khattak, S., *et al.* (2018). Single-cell analysis uncovers convergence of cell identities during axolotl limb regeneration. *Science*, page eaaq0681.

Grün, D. *et al.* (2014). Validation of noise models for single-cell transcriptomics. *Nature methods*, **11**(6), 637–640.

Guo, G. *et al.* (2010). Resolution of Cell Fate Decisions Revealed by Single-Cell Gene Expression Analysis from Zygote to Blastocyst. *Developmental Cell*, **18**(4), 675–685.

Haghverdi, L., Buettner, F., and Theis, F. J. (2014). Diffusion maps for high-dimensional single-cell analysis of differentiation data. *Bioinformatics*, **31**(18), 2989–2998.

Hashimshony, T. *et al.* (2016). CEL-Seq2: sensitive highly-multiplexed single-cell RNA-Seq. *Genome biology*, **17**(1), 77.

Hunter, J. D. (2007). Matplotlib: A 2d graphics environment. *Computing In Science & Engineering*, **9**(3), 90–95.

Islam, S. *et al.* (2014). Quantitative single-cell RNA-seq with unique molecular identifiers. *Nature methods*, **11**(1), 163–166.

Ji, Z. and Ji, H. (2016). Tscan: Pseudo-time reconstruction and evaluation in single-cell rna-seq analysis. *Nucleic acids research*, **44**(13), e117–e117.

Jones, E., Oliphant, T., Peterson, P., *et al.* (2001–). SciPy: Open source scientific tools for Python.

Parra, R. G., Papadopoulos, N., Ahumada-Arranz, L., El Kholtei, J., Treutlein, B., and Soeding, J. (2018). Reconstructing complex lineage trees from scRNA-seq data using MERLoT. *bioRxiv*.

Paul, F. *et al.* (2015). Transcriptional heterogeneity and lineage commitment in myeloid progenitors. *Cell*, **163**(7), 1663 – 1677.

Pérez, F. and Granger, B. E. (2007). IPython: a system for interactive scientific computing. *Computing in Science and Engineering*, **9**(3), 21–29.

R Core Team (2017). *R: A Language and Environment for Statistical Computing*. R Foundation for Statistical Computing, Vienna, Austria.

RStudio Team (2015). *RStudio: Integrated Development Environment for R*. RStudio, Inc., Boston, MA.

Siebert, S., Farrell, J. A., Cazet, J. F., Abeykoon, Y., Primack, A. S., Schnitzler, C. E., and Juliano, C. E. (2018). Stem cell differentiation trajectories in hydra resolved at single-cell resolution. *bioRxiv*.

Tange, O. (2011). Gnu parallel - the command-line power tool. *The USENIX Magazine*, pages 42–47.

Trapnell, C., Cacchiarelli, D., Grimsby, J., Pokharel, P., Li, S., Morse, M., Lennon, N. J., Livak, K. J., Mikkelsen, T. S., and Rinn, J. L. (2014). Pseudo-temporal ordering of individual cells reveals dynamics and regulators of cell fate decisions. *Nature biotechnology*, **32**(4), 381.

van der Walt, S., Colbert, S. C., and Varoquaux, G. (2011). The numpy array: A structure for efficient numerical computation. *Computing in Science Engineering*, **13**(2), 22–30.

van Rossum, G. (1995). Python tutorial. Technical Report CS-R9526, Centrum voor Wiskunde en Informatica (CWI), Amsterdam.

Velten, L., Haas, S. F., Raffel, S., Blaszkiewicz, S., Islam, S., Hennig, B. P., Hirche, C., Lutz, C., Buss, E. C., Nowak, D., *et al.* (2017). Human haematopoietic stem cell lineage commitment is a continuous process. *Nature cell biology*, **19**(4), 271.

Vinh, N. X., Epps, J., and Bailey, J. (2010). Information theoretic measures for clusterings comparison: Variants, properties, normalization and correction for chance. *J. Mach. Learn. Res.*, **11**, 2837–2854.

Zappia, L., Phipson, B., and Oshlack, A. (2017). Splatter: simulation of single-cell RNA sequencing data. *Genome Biology*, **18**(1), 174.

Zeisel, A. *et al.* (2015). Cell types in the mouse cortex and hippocampus revealed by single-cell RNA-seq. *Science*, **347**(6226), 1138–42.

Zhu, C., Byrd, R. H., Lu, P., and Nocedal, J. (1997). Algorithm 778: L-bfgs-b: Fortran subroutines for large-scale bound-constrained optimization. *ACM Trans. Math. Softw.*, **23**(4), 550–560.
